# Comparative Analysis of Liposuction and Conservative Treatment in Lipedema Patients: A Modified Body-Q Questionnaire Study

**DOI:** 10.3390/jcm14010279

**Published:** 2025-01-06

**Authors:** Marie-Luise Aitzetmüller-Klietz, Mahmut Ozturk, Tobias Seefeldt, Philipp Wiebringhaus, Sascha Veiz Wellenbrock, Baksan Tav, Tobias Hirsch, Kamran Harati, Matthias Aitzetmüller-Klietz

**Affiliations:** 1 Department of Plastic Surgery, University Hospital Muenster, Waldeyerstrasse 1, 48149 Muenster, Germanysascha.wellenbrock@yahoo.com (S.V.W.); baksan@gmail.com (B.T.); aitzetmueller.m@hotmail.com (M.A.-K.); 2 Department for Plastic and Reconstructive Surgery, Institute for Musculoskeletal Medicine, Westfaelische Wilhelms-University Muenster, 48149 Münster, Germany; 3 Department of Plastic, Reconstructive, and Aesthetic Surgery, Hand Surgery, Fachklinik Hornheide, Dorbaumstrasse 300, 48157 Muenster, Germany; 4 Department of Plastic, Aesthetic, Reconstructive and Hand Surgery, Klinikum Passau, Medical Campus of Lower Bavaria, 94032 Passau, Germany

**Keywords:** lipedema, liposuction, quality of life, body contouring

## Abstract

**Background**: Despite its estimated high prevalence among women and increasing awareness, lipedema remains under-investigated. Ignoring its debilitating nature, surgical treatment for this condition is frequently covered by health insurance only in advanced stages and after the exhaustion of conservative therapies. **Methods**: A total of 1015 patients with lipedema were recruited via social media platforms. Of these, 860 patients provided answers to at least one complete section of the modified Body-Q questionnaire (response rate 85%). The Mann–Whitney U and Kruskal–Wallis tests were utilized to assess the impact of the surgical treatment by means of patient-reported outcomes on the self-perception of various body areas. **Results**: The satisfaction scores among conservatively treated patients for abdominal appearance, arms, back, body, buttocks, and inner thighs showed a statistically significant decline with increasing stages of lipedema. The comparison of patient evaluation scores in the appearance domain demonstrated better patient self-perception scores in patients who received at least one session of the surgical treatment for the hips and thighs (*p* < 0.01), inner thighs (*p* < 0.01), and excess skin (0.01) scales. On the body scale, the patients who underwent liposuction again reported better satisfaction scores; however, this did not reach statistical significance (*p* < 0.081). In the health-related quality of life domain, the patients who received liposuction treatment reported a better outcome in the body image (*p* < 0.01), physical function (*p* = 0.05), physical symptoms (*p* = 0.04), and psychological function (*p* < 0.01) scales. **Conclusions**: The current study underscores the burden of lipedema of affected patients and its negative impact on self-perception. As the disease progresses, conservatively treated patients experience a decline in satisfaction with various aspects of their appearance. However, surgical interventions, particularly liposuction, does not address esthetic concerns but significantly improve health-related quality of life across multiple domains, emphasizing the comprehensive benefits of surgical intervention in the management of lipedema.

## 1. Introduction

Lipedema describes an abnormal distribution of the subcutaneous fat, predominantly in the upper and lower legs, and is attributed to hyperplasia and hypertrophy. It is distinguished from obesity and lymphedema by sparing the hands and the feet. Additionally, affected regions usually have a lower threshold for pain [1,2]. In contrast to standard weight loss methods in obesity, excess fat in lipedema is described as resistant to conventional therapies such as calorie restriction. Hence, treatment modalities such as lifestyle changes and decongestive therapy usually only provide temporary relief and are insufficient for providing a substantial improvement in quality of life for those affected. Considering the potential burden of untreated lipedema such as decreased mobility, continuous pain sensation, psychological distress, and low self-esteem, surgical interventions became inevitable.

Over the past decade, liposuction has emerged as a focal point of clinical and scientific interest as the definitive treatment modality for the relatively recently defined or acknowledged condition of lipedema over the past decade. Liposuction, which was once used primarily for esthetic reasons to remove extra fat, is now routinely used in the clinical setting in lipedema patients to alleviate the burden of the disease and improve quality of life (QoL) [3].

In this study, we sought to evaluate the impact of liposuction on quality of life and self-perception in individuals diagnosed with lipedema, with a specific focus on the elucidation of nuanced differences in self-perception and quality of life across various affected body areas and among patients with different extents of lipedema.

## 2. Materials and Methods

An anonymized, non-randomized study was conducted through March–August 2022 at the Department of Plastic Surgery, University Hospital Münster. A total of 1015 patients with medically diagnosed lipedema adhered to the online survey, which included among sociodemographic questions a modified validated and standardized version of the Body-Q [4] in German, distributed through social media. The questionnaire comprised 22 question sets (scales) with 4 to 10 questions, of which 18 were included in the modified questionnaire.

This study consisted of 40 single-choice, 4 multiple-choice, and 4 open-text questions that were directed at the participants. Additionally, participants who underwent liposuction were asked to answer questions about their post-liposuction appearance and hospitalization. Scoring was conducted on a single-item basis, and a global score was also calculated, which was transformed into an equivalent Rasch score ranging from 0 (worst) to 100 (best). The content validity and reliability of the German version of the Body-Q were tested in the original publication by A.F. Klassen et al., ‘The BODY-Q: A Patient-Reported Outcome Instrument for Weight Loss and Body Contouring Treatments’ [4]. We obtained the necessary agreement from the questionnaire developers before using the questionnaire in our study.

Of the 1015 participants, 860 provided complete answers to at least one complete section and were considered for analysis.

The participants were then grouped based on their symptoms to stages of disease and treatment modality. The stages of lipedema were defined based on the current guidelines [5] as follows:

Stage I: Smooth skin surface with an evenly enlarged and homogeneous-looking hypodermis.

Stage II: Uneven, largely wavy skin surface and nodular structures in the enlarged hypodermis.

Stage III: Considerably enlarged circumference with bulge and cuff development.

The presence of accompanying lymphedema was defined and reported as Stage IV [6]. The Mann–Whitney U test was utilized for the analysis of the means among subgroups. The Kruskal–Wallis test was used to make a comparison with more than two independent subgroups. A two-tailed *p* < 0.05 was implemented for determining statistical significance. For *p*-values < 0.05, a post hoc analysis was carried out using a pairwise Mann–Whitney U test. A *p*-value of less than 0.1 of this sub-analysis was considered significant. The statistical analysis was performed with IBM SPSS Statistics 28 (25 May 2021) and R (RStudio, 2022.07.0, Boston, MA, USA).

## 3. Results

### 3.1. Demography

Table 1 presents an overview of the sociodemographic parameters, patient characteristics, and Body-Q scales. Of all the respondents, 99% (*n*: 851) were female, and 0.2% (*n*: 2) were male. The lipedema classification of the patients was as follows: 124 were in stage 1 (14%), 523 in stage 2 (61%), 165 in stage 3 (19%), and 18 in stage 4 (2%). In total, 3% (30 patients) were unspecified.

Out of the total number of participants, 255 reported undergoing one or more liposuctions, while the remaining 605 participants received conservative treatment and served as a control group.

The participants showed an increase in the adipose tissue of the outer thighs, inner thighs, and lower legs (827 (96%), 816 (95%), and 726 (84%) retrospectively). Additionally, 716 participants (83%) mentioned a deposit of adipose tissue or lipedema-associated symptoms in the upper arms.

The number of liposuction procedures in the cohort ranged from 1 to 12, with an average of 2.7 liposuctions. A total of 66 participants (27%) received more than three liposuctions.

The exact number and regions of liposuction procedures are given in Table 1.

#### 3.1.1. Comparison of the Patients Treated with Conservative Measures

##### Appearance

Of the nine scales, the analysis of satisfaction with abdominal appearance showed a gradual decrease in median Rasch scores with increasing stages of lipedema. Between the first and second (*p* < 0.001) and the second and third (*p* < 0.001) stages, this decrease was statistically significant. However, a similar significant reduction in median Rasch scores was not observed between the third and fourth stages (*p* = 1.00).

A similar trend was present on the arms, back, body, buttocks, and inner thigh scales. The patients in the first stage of lipedema reported the highest satisfaction scores for these scales, and with each stage, the satisfaction score dropped significantly (*p* < 0.001), except for the fourth stage. The breast scale also demonstrated a significant worsening of satisfaction score; however, the correlation was of weaker evidence compared to the aforementioned scales (Stage I vs. Stage II, *p* = 0.047, and Stage II vs. Stage III, *p* = 0.014).

On the hips and outer thigh scale, a significantly lower score was observed between the first and the second stages (*p* < 0.001). Although the satisfaction scores from the patients with the third stage were lower, the comparison between the second and third stage did not reach statistical significance (*p* = 0.078).

**Figure 1:**
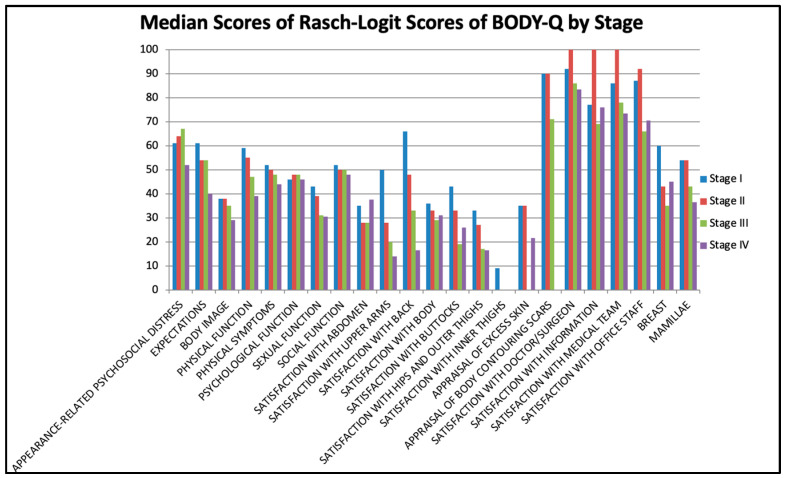
Median scores for BODY-Q scores by stages.

On the excess skin scale, the patients with lipedema stage III had a lower score than patients with stage II (*p* = 0.037); however, this difference was not observed between other stages. Lastly, the nipple scale did not reveal any difference in terms of satisfaction among patients with lipedema (Figure 1).

##### Health-Related Quality of Life

In this domain, eight scales were utilized to assess patient evaluation on health-related quality of life. Among these, on the physical function (Stage I vs. Stage II, *p* < 0.001, and Stage II vs. Stage III, *p* < 0.001) and physical symptoms (Stage I vs. Stage II, *p* < 0.001, and Stage II vs. Stage III, *p* = 0.004) scales, a significant decrease in patient scores with increasing stages of the disease was observed, except between the third and fourth stages. On the appearance-related psychosocial distress scale, gradual worsening was marked with a weaker correlation among the patients with the second and third stages of the disease (*p* = 0.056).

On the sexual function scale, there was a marked reduction in patient scores between the first and second stages (*p* = 0.001), whereas on the social function scale, this was more emphasized between the second and third stages (*p* = 0.027). On the expectations and psychological symptoms scales, there was no notable difference in the evaluation scores of patients with different stages of lipedema.

#### 3.1.2. Impact of the Surgical Treatment on Quality of Life

##### Appearance

**Figure 2:**
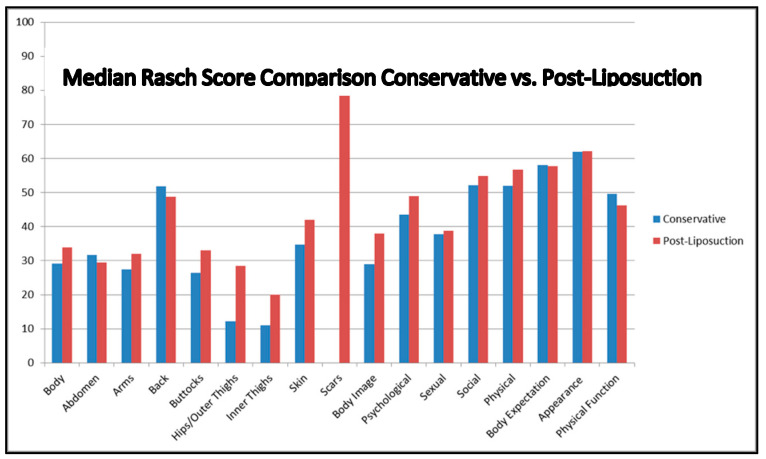
Median Rasch scores comparing conservative and surgical treatment.

The comparison of patient evaluation scores in the appearance domain demonstrated better patient self-perception scores in patients who received at least one session of the surgical treatment for the hips and thighs (*p* < 0.01), inner thighs (*p* < 0.01), and excess skin (*p* < 0.01) scales. On the body scale, the patients who underwent liposuction reported again better satisfaction scores; however, this did not reach statistical significance (*p* < 0.081) (Table 2, Figure 2). 

##### Health-Related Quality of Life

In the health-related QoL domain, the patients who received liposuction treatment reported better outcomes on the body image (*p* < 0.01), physical function (*p* = 0.05), physical symptoms (*p* = 0.04), and psychological function (*p* < 0.01) scales. On the other scales of this domain, the reported scores did not differ among surgically treated and non-treated patients (Table 3, Figure 2).

## 4. Discussion

Despite its estimated high prevalence among women and increasing awareness, lipedema remains under-investigated [7]. In daily clinical practice, it is still often not recognized or masked by accompanying adiposity [8]. Many countries or healthcare systems still either do not recognize it as a disease or recognize it to a certain extent, neglecting its physical, social, and psychological impact on affected patients. The current study delineates the self-perception of patients with different stages of lipedema who were treated either conservatively or surgically, utilizing the well-established Body-Q questionnaire to assess the patient-reported outcome.

### 4.1. Appearance and Health-Related Quality of Life in Conservatively Treated Patients

The satisfaction scores among conservatively treated patients for abdominal appearance, arms, back, body, buttocks, and inner thighs showed a statistically significant decline with stage progression, especially when comparing the first and second stage and the second and third stage. However, the decrease in satisfaction scores was not statistically significant between the third and fourth stage. A similar trend was observed on the breast scale, with a significant reduction in satisfaction scores between stages, albeit with weaker evidence compared to other scales.

The fact that the scores for the satisfaction of abdominal appearance, arms, back, body, buttocks, and inner thighs show a statistically significant decrease as lipedema proceeds demonstrates the profound impact of the illness on patients’ physical and psychological well-being. In more progressive stages of lipedema, the accruing manifestations on the body translate to an increased sense of body image and functional dissatisfaction. This trend illustrates the increasing physical load placed on patients, and hence the urgent need for early detection and management. This could be realized by diagnosing lipedema at a relatively early stage, and therefore the role of clinicians—especially in primary healthcare—being aware and alert to monitoring and treating the condition is paramount. Still, even more distressing is the noteworthy sharp decrease in patient satisfaction scores along the continuum of the disease severity of lipedema. These findings infer that with the progression of the disease of lipedema, patients are emotionally burdened more in terms of lowered self-esteem and heightened self-consciousness. These reiterate total management policies and programs necessary for the physiological and psychological impacts of the disease. Patient education, social support, and mental health resources should be addressed before improving the health and quality of life of individuals with lipedema in healthcare systems. These results encourage an entire-individual approach in the management of lipedema as physical treatment alone is not sufficient to address patients’ concerns.

The presence of significant differences in the satisfaction scores for certain body regions between stages one and two, but not between stages two and three, such as for the hips and outer thighs, may suggest the beginning of the negative impact of the disease on affected patients at earlier stages in certain body areas. The hips and outer thigh scale further demonstrated a significantly lower score between the first and second stages, but the comparison between the second and third stage did not reach statistical significance.

It was indicated that the patients notice physical and psychological changes as early as in the second stages, and by the time these reach the later stages, they are already beyond the initial stage of complaints and adjusted to the disease’s effects, resulting in a plateau in satisfaction scores.

In the HRQoL domain, the Rasch scores for the physical function and physical symptoms as well as appearance-related psychosocial distress scales, similar to the appearance domain, became worse with the increasing stage of the disease. For the sexual function scale, the difference between stage one and two was significantly worse, but not between the second and third stages. On the contrary, the impact of the disease was noticeable between the second and third stage, but not between the first and second stage on the social function scale. There was no difference among the patients with different stages of the disease on the expectation and psychosocial symptoms scales. The earlier worsening of the sexual function outcome indicates a greater impact of lipedema in patients’ private life, prior to the appearance of complaints in social life.

### 4.2. Appearance and Health-Related Quality of Life in Surgically Treated Patients

The results of the patients who underwent at least one session of surgical therapy provide a valuable input on the effectiveness of surgical intervention on different aspects of patient satisfaction and quality of life. The marked satisfaction scores observed in the domain of appearance, particularly on the hips and thighs scales, inner thigh scales, and excess skin scales, affirm the positive impact of liposuction on relieving physical symptoms as well as impairments in self-perception resulting from lipedema. The scores on the body scale also indicate better outcomes for patients who were surgically treated, but this did not reach significance (*p* = 0.081), which could indicate the need for a larger sample size for verifying this phenomenon.

Confirming the findings in the appearance domain, patients also reported significantly better evaluation scores in the body image, physical functioning, physical symptoms, and social functioning scales of the health-related quality of life domain, emphasizing that liposuction does more than reduce the physical burden of lipedema as it helps alleviate the psychosocial distress and social limitations imposed by the disease. Significantly better scores of surgically treated patients in terms of body image and physical functioning suggest that surgical intervention elevates the self-esteem and overall involvement of the patients in daily life. Due to the study design, comparison with a healthy cohort was not possible, representing a limitation.

The purpose of this study was to assess the impact of liposuction on the quality of life and physical appearance of lipedema patients using the Body-Q questionnaire. Our findings indicate that as lipedema advances, patients become increasingly dissatisfied with their appearance and body image, particularly in the hips and inner and outer thighs. A comparison between participants who underwent conservative treatment and those who underwent liposuction revealed significant improvements in the appearance and body image scores after liposuction. Moreover, liposuction positively affected both quality of life and health-related quality of life (HRQOL) scores, confirming the findings of previous studies with a validated questionnaire [1,9,10].

## 5. Conclusions

This study demonstrates the negative impact of lipedema on one’s self-perception and quality of life and the potential advantages of surgical treatment. The progression of lipedema negatively correlates with QoL and results in greater dissatisfaction with body perception, while early recognition and treatment of the disease, as well as the utilization of liposuction already in earlier stages, could be considered to alleviate the disease’s progression, as well as the disease-related physical, psychological, and social burden.

## Figures and Tables

**Table 1:** Demographic data of included patients and procedures.

Sociodemographic Parameters	*n*	%
Gender		
Female	851	99.0%
Male	2	0.2%
Uncommented	7	0.8%
Stages of lipedema		
Stage I	124	14%
Stage II	523	61%
Stage III	165	19%
Stage IV	18	2%
Uncommented	30	3%
Treatment		
Liposuction	255	30%
Conservative	597	70%
Area affected by lipedema	*n*	860
Lower leg	726	84%
Outer thighs	827	96%
Inner thighs	816	95%
Buttocks	665	77%
Upper arm	716	83%
Forearm	340	40%
Area of liposuction	*n*	255
Lower leg	188	74%
Thigh	234	92%
Buttocks	107	42%
Upper arm	126	49%
Forearm	101	40%
Abdomen	52	20%
Number of liposuctions		
1	69	28%
2	67	27%
3	46	19%
>3	66	27%
Hospitalization	Median	Range
Medical team	85	0–100
Information	76	0–100
Surgeon	84	0–100
Office staff	83	0–100

**Table 2:** Self-perception scores were better for the hips and thighs, inner thighs, and excess skin scales in patients who received at least one session of the surgical treatment. C: control group; L: liposuction group; X0.25–X0.75: quartiles; X50: median; σ: standard deviation; σ(Ⴟ): standard error; Ⴟ: mean.

Item	Treatment	*n*	X0.25	X0.5	X0.75	σ	σ(Ⴟ)	Ⴟ	Median Test	*p*	Chi-Squared
Abdomen	C	597	7	32	43	26.29	1.08	31.12	Adj. sig.	0.353	0.73
	L	255	0	28	46	26.52	1.64	28.06			
Arms	C	597	0	28	42	23.41	0.96	26.81	Adj. sig.	0.275	1.04
	L	255	0	28	50	28.18	1.74	30.26			
Back	C	597	33	54	66	31.65	1.30	50.32	Adj. sig.	0.089	2.64
	L	255	15	42	66	33.59	2.07	45.42			
Body	C	597	16	29	38	15.74	0.64	28.63	Adj. sig.	0.081	2.80
	L	255	20	33	42	19.15	1.18	32.37			
Buttocks	C	597	0	29	43	22.57	0.92	25.90	Adj. sig.	0.21	1.39
	L	255	0	33	48	27.05	1.67	30.87			
**Hips and thighs**	**C**	**597**	**0**	**0**	**22**	**16.62**	**0.68**	**11.89**	**Adj. sig.**	**<0.01**	**32.42**
	**L**	**255**	**0**	**22**	**44**	**26.83**	**1.65**	**26.71**			
**Inner thighs**	**C**	**597**	**0**	**0**	**23**	**16.70**	**0.68**	**10.55**	**Adj. sig.**	**<0.01**	**6.95**
	**L**	**255**	**0**	**0**	**33**	**25.27**	**1.56**	**18.89**			
**Excess skin**	**C**	**597**	**0**	**0**	**35**	**26.66**	**1.09**	**20.42**	**Adj. sig.**	**<0.01**	**19.00**
	**L**	**255**	**0**	**28**	**50**	**30.67**	**1.89**	**31.10**			
Scars	C	597	-	-	-	-	-	-	-	-	-
	L	255	45	83	100	38.92	2.4	67.78			
Breast	C	597	25	39	52	22.43	0.92	36.52	Adj. sig.	0.108	2.36
	L	255	25	41	60	24.69	1.52	39.89			

**Table 3:** Patients who underwent liposuction treatment reported significantly better outcomes in the health-related quality of life (QoL) domain, specifically in the areas of body image, physical function, physical symptoms, and psychological function. C: control group; L: liposuction group; X0.25–X0.75: quartiles; X50: median; σ: standard deviation; σ(Ⴟ): standard error; Ⴟ: mean.

Item	Treatment	*n*	X0.25	X0.5	X0.75	σ	σ(Ⴟ)	Ⴟ	Median Test	*p*	Chi-Squared
Appearance distress	C	597	52	61	70	17.10	0.70	61.31	Adj. sig.	0.124	2.14
	L	255	52	61	73	21.03	1.30	60.30			
**Body image**	**C**	**597**	**17**	**31**	**42**	**18.52**	**0.76**	**28.51**	Adj. sig.	**<0.01**	**13.64**
	**L**	**255**	**27**	**38**	**47**	**20.34**	**1.25**	**35.88**			
Expectations	C	597	40	54	64	23.79	0.97	51.21	Adj. sig.	0.925	0.00
	L	255	45	54	67	22.34	1.38	53.19			
**Physical function**	**C**	**597**	**39**	**50**	**62**	**19.21**	**0.79**	**51.48**	**Adj. sig.**	**0.05**	**3.56**
	**L**	**255**	**42**	**55**	**71**	**22.53**	**1.39**	**54.36**			
**Physical symptoms**	**C**	**597**	**40**	**48**	**55**	**12.49**	**0.51**	**47.66**	**Adj. sig.**	**0.04**	**7.56**
	**L**	**255**	**42**	**50**	**60**	**15.65**	**0.97**	**50.05**			
**Psychol. function**	**C**	**597**	**34**	**42**	**52**	**16.39**	**0.67**	**42.80**	**Adj. sig.**	**<0.01**	**13.61**
	**L**	**255**	**38**	**48**	**57**	**18.98**	**1.17**	**46.33**			
Sexual function	C	597	18	39	51	23.21	0.95	35.57	Adj. sig.	0.461	0.44
	L	255	26	35	51	23.44	1.45	35.54			
Social function	C	597	40	50	60	16.51	0.68	51.08	Adj. sig.	0.168	1.70
	L	255	42	50	62	18.65	1.15	51.89			

## Data Availability

The original contributions presented in this study are included in the article. Further inquiries can be directed to the corresponding author.
